# Epibulbar osseous choristoma with dermolipoma: A case report and review of literature

**DOI:** 10.1097/MD.0000000000031555

**Published:** 2022-11-25

**Authors:** Ju Mi Kim, Woo Young Son, Hae Joung Sul, Jeongah Shin, Won-Kyung Cho

**Affiliations:** a Department of Ophthalmology, Daejeon St. Mary’s Hospital, College of Medicine, The Catholic University of Korea, Seoul, Republic of Korea; b Department of Pathology, Daejeon St. Mary’s Hospital, College of Medicine, The Catholic University of Korea, Seoul, Republic of Korea; c Department of Ophthalmology Uijeongbu St. Mary’s Hospital, College of Medicine, The Catholic University of Korea, Seoul, Republic of Korea.

**Keywords:** congenital subconjunctival mass, epibulbar dermolipoma, epibulbar osseous choristoma

## Abstract

A choristoma is defined as a growth of histologically normal tissue in an abnormal location. Epibulbar osseous choristoma is the rarest type among all ocular choristoma with less than 100 cases reported. Here, we report a case of epibulbar osseous choristoma combined with dermolipoma and a literature review

**Methods:**

A 15-year-old female patient presented with an accidentally found subconjunctival mass in her left eye. Slit lamp examination revealed a 10 × 10 mm elevated, sigmoid-shaped mass in the supratemporal quadrant of the bulbar conjunctiva. We performed a debulking excisional biopsy of the mass.

**Results:**

The pathology confirmed osseous tissue surrounded by mature adipose tissue. At 1 week after the operation, the wound was clear and the patient was satisfied with the treatment. A systematic literature review of 14 previously published cases taken from PubMed dating back to 1987 along with ours was undertaken. The average age at presentation was 11.6 years and there was a female preponderance with 10 cases being female and the other 5 cases being male. Supratemporal conjunctiva was the most common site of presentation. There was no systemic disease associated with any of the cases. Since it is a benign tumor, it can be managed by observation, but if necessary, it can be treated by surgical removal.

**Conclusion:**

In pediatric subconjunctival mass, particularly located in supratemporal quadrant of bulbar conjunctiva, osseous choristoma should be considered in the differential diagnosis. Pre-operative CT scans will helpful to not also reduce complication with surgical excision but also helpful in prediction of diagnosis and prognosis.

## 1. Introduction

A choristoma is defined as a benign mass of normal tissue in an abnormal location. Predominant locations of ocular choristoma include epibulbar region, ocular adnexae, and choroid.^[1]^ Epibulbar osseous choristoma is the rarest type of all ocular choristoma with less than 100 cases reported since its first report in 1863.^[2–4]^ It is most commonly found in the supratemporal quadrant of the bulbar conjunctiva. While it is believed to be congenital, it is often found in childhood because of its asymptomatic nature and its location underneath the eyelid. It can present as a single-tissue of mesodermal or ectodermal origin or comprise 2 or more different tissues of mesenchymal and ectodermal origin. Here, we present a case of an epibulbar osseous choristoma surrounded by ectopic adipose tissue and briefly review published literature on similar cases.

## 2. Case presentation

**Figure 1.**
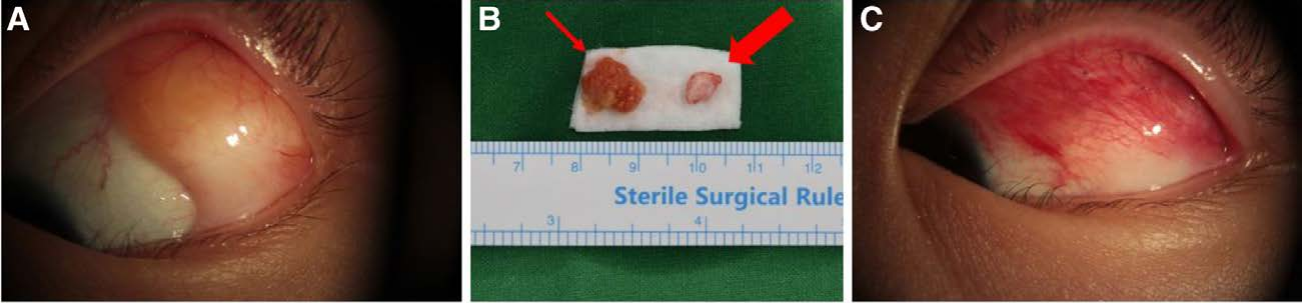
(A) Initial slit lamp photograph showing an elevated, fat-containing, sigmoid-shaped conjunctival mass of left eye. (B) Specimen collected: 7 × 5 mm osseous tissue (thick arrow) and 12 × 10 × 3 mm surrounding soft tissue (thin arrow). (C) Slit lamp photograph at 1 week after the mass excision. The visible mass at supratemporal bulbar conjunctiva was resected completely and the patient did not complain of any symptoms.

A 15-year-old female patient was referred to our hospital with an accidentally noticed conjunctival mass at the supratemporal side of her left eye. The patient denied of any underlying medical condition or ocular history. Her visual acuity was 20/20 in both eyes and intraocular pressure was normal. Slit-lamp examination revealed a 10 × 10 mm sized, elevated, yellowish fat-colored, rich in blood vessels, and sigmoid-shaped mass at supratemporal side of bulbar conjunctivae in the left eye (Fig. 1A). The superior portion of the mass had convex margin and resembled typical appearance of conjunctival fat prolapse, while the inferior portion of the mass had concave margin and showed suspicious white fibrotic change. Funduscopic examination revealed no abnormalities. Findings in the right eye were normal.

**Figure 2.**
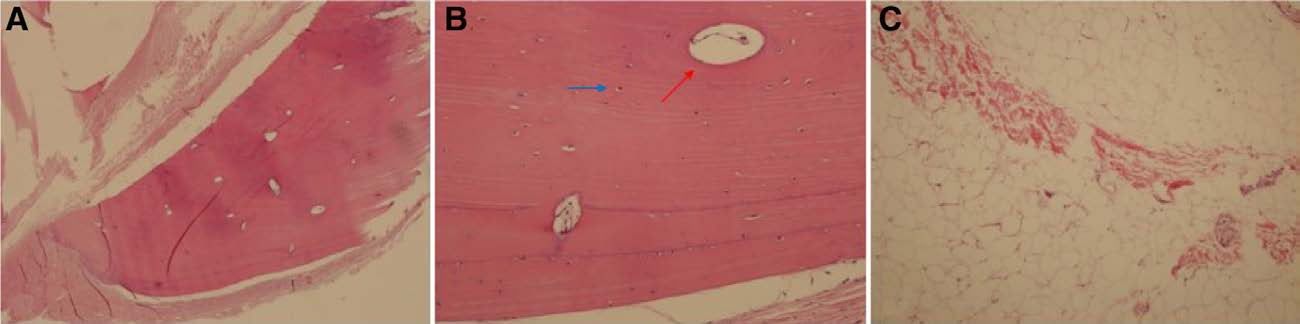
The pathologic section of the collected specimens. (A) well-circumscribed lamellar bone structure on low power magnification (hematoxylin & eosin stain, ×20 magnification). (B) Higher power magnification revealing Haversian canals surrounded by concentric rings of lamellar bone (red arrow) and osteocytes (blue arrow) (hematoxylin & eosin stain, ×400 magnification). (C) Lobular adipose tissue (hematoxylin & eosin stain, ×20 magnification).

For diagnostic and cosmetic purposes, we performed debulking excision of the mass. Under local anesthesia, the mass at the supratemporal bulbar conjunctiva was exposed with speculum. We noticed whitish fibrous band at inferior portion of the mass looking more evident under microscope. Mass was demarcated and incision line was drawn with a marking pen. 2% lidocaine and 1:100,000 epinephrine mixed solution was injected. Conjunctival incision was made with Westcott scissors. Subconjunctival tissue was carefully dissected while controlling bleeding. We identified a hard, bone-like mass surrounded by a thick fibrous band embedded within an overlying fat tissue. We completely excised the visible mass and 2 specimens were collected separately for biopsy: bony tissue with size of 7 × 5 × 2 mm and surrounding fatty tissue with size of 12 × 10 × 3 mm (Fig. 1B). After bleeding control, the conjunctiva was sutured with absorbable suture 8/0 Vicryl (Ethicon). Histopathology confirmed osseous choristoma consisting of cortical lamellar bone with Haversian canals surrounded by thin layers of connective tissue and overlying adipose tissue (Fig. 2). At 1 week after the operation, the wound was clear and the patient did not complain of any discomfort (Fig. 1C).

## 3. Discussion

Choristoma, a histologically normal tissue expressed in an abnormal location, represents 10% to 33% of conjunctival lesions in children.^[5]^ It can be divided according to 4 histopathological findings: dermoid, dermolipoma, single-tissue choristoma, and complex choristoma.^[1]^ A dermoid is a benign tumor composed of dermal appendages and adnexal structures such as hair follicles and sebaceous glands. A dermolipoma is a congenital choristoma characterized by an abnormal epibulbar growth of mostly adipose tissue. It is distinguished from orbital fat prolapse, which is defined as a herniation of intraconal orbital fat due to weakened Tenon’s capsule by aging process, trauma, or surgery. While an orbital fat prolapse appears mostly as a yellow, soft, mobile mass with convex anterior border, a dermolipoma is a pink-yellow, soft, non-mobile mass with straight or slightly concave anterior border.^[6]^ Dermoid and dermolipoma are the most common choristomas that present in the conjunctiva.^[7]^ Sometimes they can present in association with systemic diseases such as Goldenhar’s syndrome, encephalocraniocutaneous lipomatosis, and linear nevus sebaceous syndrome.^[7]^ Single-tissue choristoma contains an ectopic tissue of mesodermal origin such as bone, cartilage, muscles, and connective tissue or ectodermal origin like cutaneous appendage, nerve, brain, and lacrimal gland.^[8]^ Complex choristoma includes a combination of tissues of both origins.^[8]^ Choristoma should not be confused with hamartoma and teratoma. Hamartoma is a benign, tumor-like proliferation of abnormal cells and tissues found where the tissue normally grows. In contrast, choristoma is a benign mass made up of normally developed tissues found at sites other than its normal area. Teratoma is defined as a germ cell tumor that contains mature and/or immature cells originating from more than 1 primitive germ cell layer.

Epibulbar oseeous choristoma, first described by Von Graefe in 1863, is the rarest type of ocular choristoma with less than 100 cases reported worldwide.^[2]^ Histological finding of epibulbar osseous choristoma typically shows mature well-circumscribed lamellar bone structure with Haversian canal system surrounded by dense, fibrous connective tissue.^[9,10]^ While it occurs most commonly in supratemporal subconjunctival space attached to or separate from sclera, it can also be found at various locations such as perilimbal area, lateral canthus, extraocular muscle insertions, and tarsal plate.^[11–14]^

Some explanations for the occurrence of epibulbar osseous choristoma have been proposed. Duke Elder first proposed the existence of secondary ossification centers of the orbital bones which very rarely could remain isolated, giving rise to small bones identified as osseous choristoma.^[15]^ Others proposed that epibulbar osseous choristoma represents an atavistic remnant of scleral ossicles that can be seen in lower vertebrates.^[9]^ However, its consistently reported location in the supratemporal region near the zygomaticofrontal suture and in the episcleral or conjunctiva and not in the sclera most likely represents an embryonal development anomaly which leads to an accessory ossicle as a result of a closure defect.^[16]^

We conducted a systematic literature review on previously reported cases via PubMed using the keywords “congenital subconjunctival mass,” “epibulbar osseous choristoma” and “epibulbar dermolipoma.” Full-text, free-access, English-printed articles with pathologically confirmed epibulbar osseous choristoma and dermolipoma identified 14 cases (Table 1).^[8,11–13,17–27]^ Including our case, age range at presentation was from birth to 38 years with mean age of 11.6 years. Ten cases (66.7%) were females, and 5 cases (33.3%) were males. This female preponderance of epibulbar choristomas has been reported in previous literature review by Gayre et al (69% of epibulbar osseous choristoma in females vs 31% in males) and Vachette et al (59% of epibulbar choristoma in females vs 41% in males). Eight lesions (53.3%) were found in right eye, and 7 lesions (46.7%) were found in left eye. None of the cases had associated systemic diseases. The lesions were found in the supratemporal (10 cases, 66.7%), lateral canthal (4 cases, 26.7%), and inferotemporal (1 case, 6.6%) areas. One case which appeared in inferotemporal area extended to limbus. While 10 of the 14 previously reported cases and our case revealed just bone and dermolipoma on histopathology, the other 4 cases included tissues other than fatty tissue and bone indicative of complex choristoma.

**Table 1:** Review of cases of epibulbar osseous choristoma with a dermolipoma.

Case no.	Authors	Age, yr	Sex	Eye	Location	Management	Tissue elements	Pre-operative CT	Pre-operative Ultrasound
1	Wang et al^[17^	23	F	OS	ST bulbar conjunctiva	Excision	Mature bone with adipose tissue	Yes	Yes
2	Lee et al^[18]^	38	F	OD	ST bulbar conjunctiva	Excision	Mature bone with adipose tissue	Yes	No
3	Herdiana et al^[8]^	18	F	OS	ST bulbar conjunctiva	Excision	Adipose tissue surrounded by bone and skeletal muscle	Yes	No
4	Ganesh and Rangaswamy^[19]^	6m	F	OS	Lateral canthus	Excision	Dermolipoma with bone, multiple nerves, smooth muscle, and skeletal muscle	No	No
5	Lissner and Bryar^[20]^	14m	F	OS	Lateral canthus	Excision	Dermolipoma with bone and hematopoetic bone marrow in the center	Yes	No
6	Hsia et al^[11]^	1m	M	OD	IT bulbar conjunctiva involving limbus	Excision	Mature bone with adipose tissue	No	No
7	Kadasi et al^[21]^	1d	F	OD	ST bulbar conjunctiva extending to lateral canthus	Excision	Mature bone with fetal adipose tissue	No	No
8	Shields et al^[22]^	14	F	OD	ST bulbar conjunctiva	Excision	Mature bone with adipose tissue	Unknown	Unknown
9		35	M	OS	ST bulbar conjunctiva	Excision	Mature bone with adipose tissue	Unknown	Unknown
10		23	F	OD	ST bulbar conjunctiva	Excision	Mature bone with adipose tissue	Unknown	Unknown
11	Tsai et al^[23]^	8	M	OS	ST bulbar conjunctiva	Excision	Mature bone with adipose tissue	Yes	No
12	Khan et al^[12]^	2	M	OD	Lateral canthus	Excision	Mature bone with adipose tissue	No	No
13	Casady et al^[24]^	18m	M	OD	Lateral canthus	Excision	Irregular collagen similar to dermis with adipose tissue and mature lamellar bone inside	No	No
14	Hered and Hiles^[25]^	6m	F	OD	ST bulbar conjunctiva	Excision	Dermolipoma with mature bone	No	No

CT = computed tomography, F = female, IT = inferotemporal, M = male, m = months, OD = oculus dexter, OS = oculus sinister, ST = superotemporal, yr = years.

In general, epibulbar osseous choristoma can be managed by observation since it is mostly asymptomatic and malignant transformation has never been reported in the literature.^[27]^ In some cases where it brings recurrent conjunctival hyperemia or ocular foreign body sensation, surgical excision may be indicated.^[3]^ Surgical excision of a mass located at supratemporal side requires particular attention not to injure adjacent superior and lateral rectus muscles and lacrimal gland ductile. Once surgical excision is planned, pre-operative computed tomography (CT) can be helpful in determining whether the mass involves nearby structures such as sclera or extraocular muscles. Futhermore pre-operative CT will not only reduce unexpected complication with surgical excision but also helpful in prediction of diagnosis and prognosis. In review of literature, CT images of epibulbar osseous choristoma revealed a cystic mass with fat-like low-density and a high-density radio-opaque mass which is indicative of a calcium component.^[8,11,12,17–24,26]^ This differentiates epibulbar osseous choristoma from low-density lesions like fat, dermolipoma, or epibulbar dermoid and helps predict the diagnosis.

In conclusion, epibulbar osseous choristoma combined with dermolipoma are rare conditions. However, 1 should consider its diagnosis when the mass is located in supratemporal subconjunctival space. Since it is a benign tumor, epibulbar osseous choristoma can be managed by observation, but if necessary, it can be treated by surgical removal, and pre-operative CT will helpful.

## Author contributions

**Conceptualization:** Ju Mi Kim, Won-Kyung Cho.

**Data curation:** Woo Young Son, Hae Joung Sul, Won-Kyung Cho.

**Formal analysis:** Jeongah Shin, Won-Kyung Cho.

**Methodology:** Ju Mi Kim, Won-Kyung Cho.

**Project administration:** Won-Kyung Cho.

**Supervision:** Jeongah Shin, Won-Kyung Cho.

**Validation:** Won-Kyung Cho.

**Visualization:** Won-Kyung Cho.

**Writing – original draft:** Ju Mi Kim, Won-Kyung Cho.

**Writing – review & editing:** Woo Young Son, Jeongah Shin, Won-Kyung Cho.
